# Editorial: Ethological dynamics in diorama environments

**DOI:** 10.3389/fcell.2023.1347957

**Published:** 2024-01-05

**Authors:** Toshiyuki Nakagaki, Audrey Dussutour, Laurence Wilson, Takuji Ishikawa

**Affiliations:** ^1^ Research Institute for Electronic Science, Hokkaido University, Sapporo, Hokkaido, Japan; ^2^ UMR5169 Centre de Recherches Sur La Cognition Animale (CRCA), Toulouse, Midi-Pyrénées, France; ^3^ Université Toulouse III Paul Sabatier, Toulouse, Occitanie, France; ^4^ School of Physics, Engineering and Technology, University of York, York, United Kingdom; ^5^ Department of Biomedical Engineering, Graduate School of Biomedical Engineering, Tohoku University, Sendai, Japan

**Keywords:** cell movement, biomechanics, protist, bio-fluid dynamics, cell behavior, sperm, cilia a flagella, collective motion

## Introduction to the scope of this Research Topic

Exploring behavioral smartness of single cells in their natural habitats is interesting as many such habitats are highly variable in space and time. We can observe that ciliates typically move around the complicated shape (in the scale of their body length) of sedimentary materials on pond beds, and these in turn are dynamic environments: water flows; cells encounter both predators and prey; and temperature, chemicals and light levels all fluctuate. Hence, the environment where they are living is complex, and they need to behave responsively.

Environmental responses are often highly adapted and pragmatic, even when the environment appears bafflingly complex to the human eye. The behavioral complexity of single cells are often unknown or ignored, while unicellular organisms express subtle and nuanced responses. Here we throw light on the behavioral abilities of single cells, and begin to unpick their underlying mechanisms.

For such studies, we propose a few key points of methodology. The first point is to well-design an experimental setup of a complex environment. We name such artificial environments as ‘diorama environment’, where organisms can show their potential abilities. Diorama environments, for example, may mimic some of the complexity of natural habitats, or may be a sequence of experimental stimulation that is designed to test the ability of learning including habituation and conditioning. The second point is to formulate a mathematical (mechanical) model to describe the smart behavior in its complex environment. The third point is, if possible, to extract the algorithm (or heuristics) of information processing for the behavior under focus. The term ‘ethological dynamics’ we propose here refers to the model equations that involve algorithms of cellular information processing for smart behavior. As the Research Topic described here is in its infancy, we hope that this Research Topic will be recognized and shared more widely in the future.

## Editorial summary of fifteen contributed papers

Cell movement can be roughly divided into two types: swimming by cilia and crawling by pseudopodia. We begin with ciliary swimming. A typical adaptive behavior observed in a diorama environment is ‘switching of behavioral modes and their modulation by a geometrical cue in the ciliate *Stentor coeruleus*’ (Echigoya et al.). In this study, designing a swimming space with a specific shape revealed *Stentor*’s ability to distinguish the space shapes. Spatial inhomogeneity is common nature of environment and, in a heterogeneous light environment, ‘long-time behavior of swimming *Euglena gracilis*’ was reported (Muku et al.).

The regulation of ciliary beating that leads to ciliary swimming are being studied as Shiba et al. reported that ‘Calaxin is required for asymmetric bend initiation and propagation in sperm flagella’, and Kijima et al. reported that ‘CatSper mediates not only chemotactic behavior but also the motility of ascidian sperm’. Based on an understanding about molecular mechanism, a mechanical model of ciliary swimming can be formulated. Ishimoto et al. investigated ‘squirmer hydrodynamics near a periodic surface topology’. Microbial hydrodynamics is a necessary tool for elucidating mechanisms for adaptive behavior in swimming microorganisms.

Regarding amoeboid movement, Nomura et al. reported a remarkable ability of pseudopod movement: ‘three-dimensional architecture and assembly mechanism of the egg-shaped shell in testate amoeba *Paulinella micropora*’. This paper shows that the pseudopods are able to complete the complex operating procedures that construct the amoeba’s shell.

The possibility of learning and memory in a single cell has been the subject of debate since many years ago, and Yoneoka et al. reported ‘relation between learning process and morphology of transport tube network in plasmodium of *Physarum polycephalum*’.

In free locomotion of *Naegleria gruberi*, ‘random walk and cell morphology dynamics’ in the absence of directional cues was characterized by a detailed and careful analysis (Uwamichi et al.), deepening our understanding of intrinsic movements that help to formulate amoeboid movement. Sato reported ‘a cell membrane model that reproduced cortical flow-driven cell migration and collective movement’. This simplified and elegant model recreates observed mechanical behavior without the need for detailed assumptions about the cell biology.

Next, we pay attention to collective motions of cells that are common in natural habitats. One of the most impressive phenomena is the algal ‘red tide’; Shikata et al. reported that ‘strain, cell density, and nutrient condition affect patterns of diurnal vertical migration and superoxide production in a red-tide alga’. At a smaller scale in the laboratory, similar collective motions called bio-convection were studied in heterogeneous environments. Rich patterns of convection spots were found in micro-alga under non-uniform light or periodic illumination, described in ‘emergence of a *Euglena* bioconvection spot controlled by non-uniform light’ (Yamashita et al.), and ‘bioconvection pattern of *Euglena* under periodic illumination’ (Suematsu et al.).

Collective swimming can be observed during fertilization in mammals when a large amount of sperm swims in the mucus layer within the reproductive tract. Phuyal et al*.* focused on ‘biological benefits of collective swimming of sperm in a viscoelastic fluid’.

Intermediate crowdedness - more than single cell and less than collective motion - is found when many species of reef-building coral show simultaneous spawning and ejaculation at a once-a-year event of their life cycle. In there, very many sperms and eggs move up to the surface of water and the sperms swim toward one of eggs under the perturbation of water waves. To initiate study on the behavior of sperm in this environment, Morita et al. reported, at a first step of study, that ‘positive selection on ADAM10 builds species recognition in the synchronous spawning coral *Acropora*’.

In multicellular organisms, the optimal foraging ability of fungal mycelial networks is clearly demonstrated in a diorama environment as Fukasawa and Ishii reported in ‘foraging strategies of fungal mycelial networks: responses to quantity and distance of new resources’.

We hope that these fifteen contributed papers will be helpful in understanding the ethological dynamics in diorama environments, and that more research on this Research Topic will be conducted in the future.

